# Five Ectopic Teeth in the Maxillary Sinus: A Rare Cause of Chronic Sinusitis

**DOI:** 10.7759/cureus.22204

**Published:** 2022-02-14

**Authors:** Mohamad Ali Ibrahim, Said EL Orra, Nagham Ramadan, Ahmad Lakis, Mohammed Dabbous

**Affiliations:** 1 Medicine, Faculty of Medicine, Saint Joseph University, Beirut, LBN; 2 Internal Medicine, Beirut Arab University, Beirut, LBN; 3 Radiology, Medical Care Laboratories and Radiology Center, Chtoura, LBN; 4 Internal Medicine, Lebanese University Faculty of Medicine, Beirut, LBN

**Keywords:** case report, supernumerary teeth, sinusitis, maxillary sinus, ectopic teeth

## Abstract

Around 10-20% of sinusitis have a dental etiology. Odontogenic sinusitis is generally caused by periodontitis, peri-implantitis, periapical pathology, or oroantral communication. Ectopic teeth are a rare cause of chronic odontogenic sinusitis. We present a rare case of chronic sinusitis caused by five ectopic teeth.

A 39-year-old-female patient presented to our clinic with complaints of facial pain over the left cheek, ipsilateral nasal obstruction, ipsilateral rhinorrhea, and coughing over the last five years. Physical examination revealed a febrile patient. There was an ipsilateral purulent nasal discharge of yellow color. Inspection of the oral cavity revealed the absence of the following maxillary teeth: left first and second premolars, in addition to the left first, second, and third molars. There was also tenderness upon palpation of the left maxillary sinus. Computed tomography (CT) scan of the maxillary sinus revealed hyperdense structures in the left maxillary sinus surrounded by soft tissue, representing the missing premolar and molar teeth. The patient was treated with amoxicillin-clavulanate and corticosteroid, which partially relieved her symptoms.

Our case presents an unusual case of chronic sinusitis that was found to be a consequence of five ectopic teeth in the maxillary sinus. A careful physical examination and an appropriate imaging modality are indispensable for the diagnosis of such a rare phenomenon.

## Introduction

Eruption of teeth is the process by which teeth migrate from the loci of their formation in the mandible and maxilla to their functional place in the oral cavity [1]. The process of teeth development starts early at the fifth week during the embryonic period [2]. Tooth maturation was found to be strongly correlated to tooth eruption timing [3]. In addition, a number of growth factors, genes, and extracellular molecules, along with the interaction between the mesenchyme and ectoderm, represent important factors for proper tooth development [4].

Teeth can sometimes reside in unusual locations; this is known as ectopic teeth eruption. The mandibular condyle, coronoid process, orbit, palate, nasal cavity, and the maxillary sinus were the predilection sites for ectopic teeth eruption as reported in the literature [5-10]. Moreover, most of the reported cases in the literature involved only one ectopic tooth, and cases reporting more than two ectopic teeth were rare [11].

To our knowledge, this is the first case of chronic sinusitis caused by five ectopic teeth in the maxillary sinus.

## Case presentation

A 39-year-old woman presented to our clinic with complaints of facial pain over the left cheek, ipsilateral nasal obstruction, ipsilateral rhinorrhea, and coughing over the last five years. Due to financial reasons, the patient did not seek medical attention and decided to manage her symptoms through antibiotics, without showing any sign of improvement. The patient denied a history of trauma. She had no relevant family history or had any surgical or dental procedures in the past.

Physical examination revealed a febrile patient (38.5°C). There was an ipsilateral purulent nasal discharge of yellow color. Inspection of the oral cavity revealed the absence of the following maxillary teeth: left first and second premolars, in addition to the left first, second, and third molars. There was also tenderness upon palpation of the left maxillary sinus.

**Figure 1:**
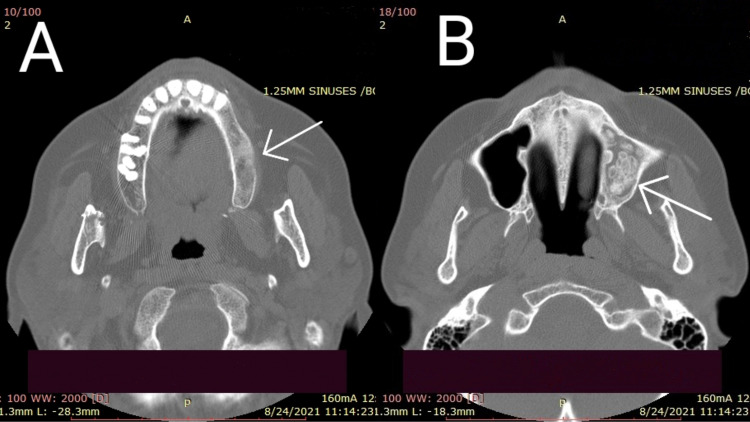
CT scan of the maxilla (A) Axial view showing the absence of the molar and premolar teeth on the left side compared to the right side, which showed normal growth of teeth. (B) Axial view showing hyperdense structures in the left maxillary sinus surrounded by soft tissue, representing the missing left premolar and molar teeth. CT, computed tomography

**Figure 2:**
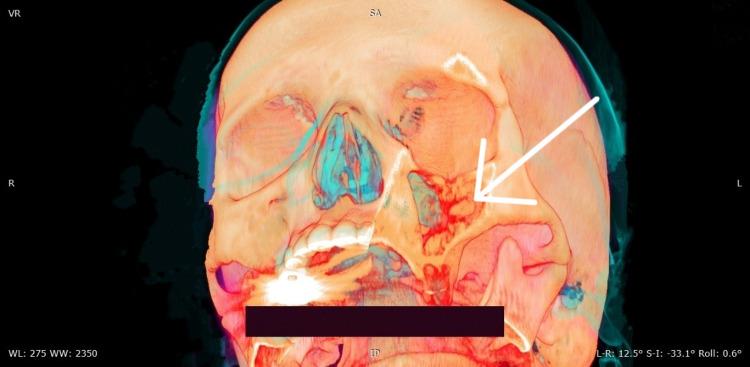
Reconstructed 3D CT image with supralateral angulation showing the undescended teeth in the left maxillary sinus 3D CT, three-dimensional computed tomography

Due to the chronicity of her symptoms and the unexplained absence of the aforementioned teeth, a CT scan of the maxillary sinus was performed, which revealed hyperdense structures surrounded by soft tissue in the left maxillary sinus, which most probably represented the missing left molar and premolar teeth (Figures 1, 2).

Due to financial reasons, tooth extraction surgery could not be performed, and the patient was instead treated with amoxicillin/clavulanic acid (875 mg/125 mg) one tablet every 12 hours for 14 days, paracetamol 1g 1 tablet every six hours, intranasal corticosteroid, and saline irrigation of the nasal cavities. Follow-up every two weeks for a period of four months revealed minimal relief of the ipsilateral facial pain, with a complete resolution of fever, ipsilateral rhinorrhea, nasal obstruction, and cough.

## Discussion

Ectopic teeth and supernumerary teeth account for 1% of the total population [12]. The etiology of ectopic tooth remains unclear. However, several factors may be implicated in its development, such as genetic factors, anomalies, trauma, and infection [13]. In our case, the patient did not have a history of trauma, crowded teeth, or any other causative factor, and thus the reason behind the ectopic teeth in our case remained unknown.

Around 52 cases of ectopic teeth in the maxillary sinus have been reported. According to multiple case reports, ectopic teeth involved the right and left maxillary sinuses equally. In addition, the third molar was found to be the most common ectopic tooth in the maxillary sinus [11].

Patients with ectopic teeth in the maxillary sinus are mostly asymptomatic, and the condition is incidentally diagnosed during an investigation for another purpose [14]. However, most reported cases described the presence of symptoms such as facial numbness, purulent nasal discharge, rhinorrhea, headache, and fever [11]. In our case, the patient presented with fever, facial pain, ipsilateral rhinorrhea, ipsilateral nasal obstruction, and coughing over five years, for which she did not seek medical attention due to the lack of financial means.

Since teeth are radiopaque, plain radiographs, panoramic radiographs, and Water graphy have the capability to detect the ectopic ones. Cone-beam computed tomography (CBCT) and CT scan are also accepted as imaging modalities for diagnosing ectopic teeth [15]. However, CT (CBCT or CT scan) remains the most accurate imaging modality, with an advantage of CBCT due to its lower dose when compared to the CT scan [16]. Due to the unavailability of CBCT in the institution, the method used was CT scan, which revealed hyperdense structures at the level of the left maxillary sinus.

A clinical follow-up is recommended when the patient is asymptomatic [11]. In symptomatic patients, the recommended treatment is surgical resection of the ectopic teeth. The Caldwell-Luc approach, with or without transoral endoscopy, and nasal endoscopy are two accepted surgical methods [17]. Surgical intervention was an indication in our case, but it was not performed due to financial reasons. However, the patient showed some improvement in her symptoms with the medical therapy.

## Conclusions

Ectopic tooth is an unusual cause of chronic sinusitis. As described in our case, the patient had chronic sinusitis over the last five years, which was unexpectedly discovered to be caused by ectopic teeth. Hence, physicians and dentists are encouraged to consider the possibility of the presence of ectopic teeth in the maxillary sinus in patients with chronic sinusitis by focusing on a careful physical examination and utilizing a suitable imaging modality.
